# Widespread Infection with Hemotropic Mycoplasmas in Free-Ranging Dogs and Wild Foxes Across Six Bioclimatic Regions of Chile

**DOI:** 10.3390/microorganisms9050919

**Published:** 2021-04-24

**Authors:** Sophia Di Cataldo, Aitor Cevidanes, Claudia Ulloa-Contreras, Irene Sacristán, Diego Peñaloza-Madrid, Juliana Vianna, Daniel González-Acuña, Nicole Sallaberry-Pincheira, Javier Cabello, Constanza Napolitano, Ezequiel Hidalgo-Hermoso, Gerardo Acosta-Jamett, Javier Millán

**Affiliations:** 1 PhD Program in Conservation Medicine, Facultad de Ciencias de la Vida, Universidad Andres Bello, República 252, Santiago 8320000, Chile; 2 Facultad de Ciencias de la Vida, Universidad Andres Bello, República 440, Santiago 8320000, Chile; aitorcevi@gmail.com or isacristan.vet@gmail.com (I.S.); syngamustrachea@hotmail.com (J.M.); 3 Department of Animal Health, NEIKER-Basque Institute for Agricultural Research and Development, Basque Research and Technology Alliance (BRTA), Parque Científico y Tecnológico de Bizkaia P812, 48160 Derio, Spain; 4 Facultad de Ciencias Veterinarias y Pecuarias, Universidad de Chile, Santa Rosa 11735, La Pintana, Santiago 8320000, Chile; clau.ulloa.c@gmail.com; 5 Parque Safari Chile, Ruta H-30, Km 5, Camino A Doñihue S/N, Rancagua, O’Higgins 2820000, Chile; dapm91@gmail.com; 6 Departamento de Ecosistemas y Medio Ambiente, Facultad de Agronomía e Ingeniería Forestal, Pontificia Universidad Católica de Chile, Av. Vicuña Mackenna 4860, Santiago 8320000, Chile; jvianna@uc.cl; 7 Departamento de Ciencias Pecuarias, Facultad de Medicina Veterinaria, Universidad de Concepción, Víctor Lamas 1290, Chillán 4070386, Chile; dgonzalezacuna@gmail.com; 8 Unidad de Rehabilitación de Fauna Silvestre, Escuela de Medicina Veterinaria, Facultad de Ciencias de la Vida, Universidad Andres Bello, República 252, Santiago 8320000, Chile; nicole.sallaberry@unab.cl; 9 Facultad de Medicina Veterinaria, Universidad San Sebastián, Puerto Montt 5480000, Chile; javier.cabello@uss.cl; 10 Departamento de Ciencias Biológicas y Biodiversidad, Universidad de Los Lagos, Av. Fuchslocher 1305, Osorno 5290000, Chile or cnapolit@uchile.cl; 11 Instituto de Ecología y Biodiversidad, Santiago 7750000, Chile; 12 Conservation and Research Department, Parque Zoológico Buin Zoo, Panamericana Sur Km 32, Buin 9500000, Chile; ezequielhidalgovet@yahoo.com; 13 Instituto de Medicina Preventiva Veterinaria y Programa de Investigación Aplicada en Fauna Silvestre, Facultad de Ciencias Veterinarias, Universidad Austral de Chile, Casilla 567, Valdivia 5091000, Chile; gerardo.acosta@uach.cl; 14 Instituto Agroalimentario de Aragón-IA2 (Universidad de Zaragoza-CITA), Miguel Servet 177, 50013 Zaragoza, Spain; 15 Fundación ARAID, Avda. de Ranillas, 50018 Zaragoza, Spain

**Keywords:** Canidae, chilla, culpeo, *Mollicutes*, South America

## Abstract

Blood samples of 626 rural dogs, 140 Andean foxes (*Lycalopex culpaeus*), and 83 South American grey foxes (*L. griseus*) from six bioregions of Chile spanning 3000 km were screened for *Mycoplasma* DNA by conventional PCR and sequencing. Risk factors of infection were inferred using Generalized Linear Mixed Models and genetic structure by network analyses. Overall, *Mycoplasma haemocanis*/*Mycoplasma haemofelis* (*Mhc*/*Mhf*) and *Candidatus* Mycoplasma haematoparvum (*C*Mhp) observed prevalence was 23.8% and 12.8% in dogs, 20.1% and 7.2% in Andean foxes, and 26.5% and 8.4% in grey foxes, respectively. Both hemoplasmas were confirmed in all the bioregions, with higher prevalence in those where ticks from the *Rhipicephalus sanguineus* species group were absent. *Candidatus* M. haematominutum and a *Mycoplasma* sp. previously found in South American carnivores were detected in one fox each. Although the most prevalent *Mhc/Mhf* and *C*Mhp sequence types were shared between dogs and foxes, network analysis revealed genetic structure of *Mhc/Mhf* between hosts in some regions. Male sex was associated with a higher risk of *Mhc/Mhf* and *C*Mhp infection in dogs, and adult age with *C*Mhp infection, suggesting that direct transmission is relevant. No risk factor was identified in foxes. Our study provides novel information about canine hemoplasmas with relevance in distribution, transmission routes, and cross-species transmission.

## 1. Introduction

Hemoplasmas are bacteria that parasitize red blood cells of a variety of domestic and wild mammals [1,2], causing acute and chronic hemolytic anemia in dogs. The infection outcome ranges from asymptomatic or slight lethargy to oncogenesis or death, depending on host susceptibility and coinfection with other pathogens [3,4]. Molecular techniques allowed the differentiation of two species of hemoplasmas in canids: *Mycoplasma haemocanis* (*Mhc*), and *Candidatus* M. haematoparvum (*C*Mhp) [5]. In addition, other hemoplasma species have been detected in dogs, such as *Candidatus* M. haemobos [6], *Candidatus* M. haematominutum [7], and *M. turicense* [8]. The transmission routes of hemoplasmas are still under debate, although blood-sucking arthropods have often been implicated in the transmission of some of these agents [9].

Information about the presence and distribution of these agents in Chile is scarce. DNA of both *Mhc* and *C*Mhp have been reported in dogs from southern Chile [10,11]. Both agents have also been detected in a wild canid, the endangered Darwin’s fox (*Lycalopex fulvipes*) [11,12]. In that study, Di Cataldo et al. [11] reported that *Mhc* was shared among dogs and foxes, but intraspecific transmission was predominant in the fox population.

Encounters between domestic animals and wildlife have become more frequent in the last years, due to the human encroachment in natural habitats, what have led to the emergence of infectious diseases [13]. The abundance of domestic dogs and their introduction into natural areas favor spillovers between closely related species [14,15]. In Chile, an estimated over four million dogs [16] are commonly free-ranging and lack sanitary care [17,18]. Free-ranging dogs often overlap habitats with other two widely distributed native foxes, the Andean fox (*Lycalopex culpaeus*) and the South American grey fox (*L. griseus*) [19,20], which can facilitate cross-species pathogen transmission.

Chile’s 4300 km length confers a bioclimatic gradient that provides an ideal scenario to study vector-borne pathogens [21,22], and can help to shed light into the transmission routes of hemoplasmas. This fact, added to the insufficient knowledge of the prevalence and distribution of hemoplasmas in dogs in the country, its presence in two abundant wild canids, and the potential interspecific hemoplasma transmission between rural dogs and wild canids, called for a focused epidemiological survey. Our goal was to describe the geographical distribution, prevalence, risk factors of infection, and potential cross-species transmission of canine hemoplasmas in dogs and foxes along the Chilean geography.

## 2. Materials and Methods

### 2.1. Study Area and Sampling Methods

**Figure 1:**
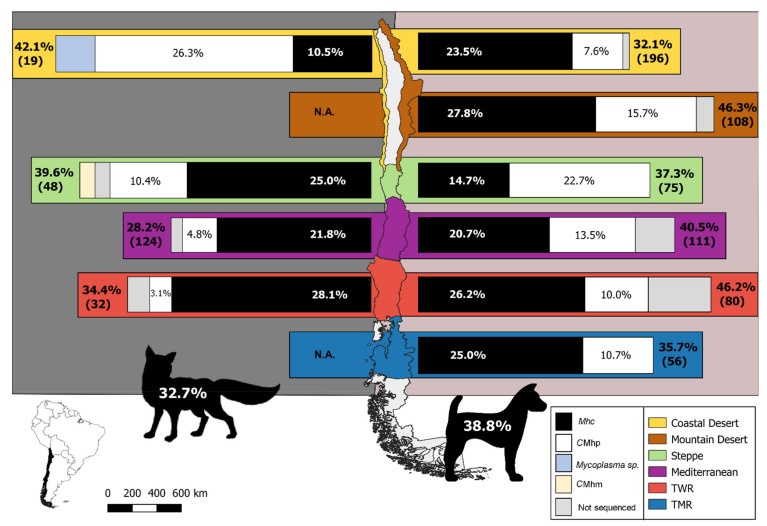
Map of the study areas, showing hemoplasma prevalence in the surveyed foxes (left) and rural dogs (right) across the different bioclimatic regions of Chile. Sample size is in parenthesis. N.A.: Not Assessed, *Mhc*: *Mycoplasma haemocanis*, *C*Mhp: *Candidatus* Mycoplasma haematoparvum, *C*Mhm: *Candidatus* Mycoplasma haematominutum, TWR: Temperate Warm Rainy, TMR: Temperate Maritime Rainy.

Six continental bioclimatic areas of Chile [23] were considered in this study, namely, from north to south: Coastal Desert, Mountain Desert, Steppe, Mediterranean, Temperate Warm Rainy (TWR) and Temperate Maritime Rainy (TMR) (Figure 1). Their distinctive bioclimate characteristics are presented in the Supplementary Table S1.

Between 2015 and 2018, 626 rural dogs were sampled with the consent of their owners. Prerequisites to be included in the study were to not have travelled far from the village where they were sampled, being free-ranging (i.e., not having permanent confinement) and not receiving any antiparasitic treatment during the last year. Dogs were classified as juveniles (less than a year) or adults (older than a year). Individual sex, year, and sampling location were recorded. Entire blood was obtained by venipuncture of the cephalic vein and collected in EDTA tubes. Ectoparasites were collected through a 5-min examination protocol [24] and stored in 90% ethanol until identification.

Eighty-three South American grey foxes (*Lycalopex griseus*) and 140 Andean foxes (*L. culpaeus*) were sampled between 2006 and 2018, either by active or passive surveillance. For active sampling, leg-hold traps (Oneida Victor Soft Catch No. 1.5, Cleveland, Ohio, USA) or tomahawk-like live traps baited with tuna or chicken were used, and foxes were anaesthetized following previous published protocols [25,26]. Passive surveillance (25 grey foxes and 60 Andean foxes) included foxes admitted to animal rehabilitation centers or road-killed animals. Due to logistic impediments, only blood was analyzed from the foxes. Whenever possible, all foxes were classified as juveniles (less than a year) or adults (older than a year) based on teeth eruption [27], and sex was recorded.

All procedures were conducted with consideration of animal welfare protocols, according to the approval of the authorities in bioethics from the Universidad Andres Bello under authorization 08/2016. Capture permits were granted by the Servicio Agrícola y Ganadero (SAG) of Chile.

### 2.2. DNA Extraction and Molecular Detection

DNA was extracted from entire blood using a DNeasy blood and tissue kit (DNeasy Blood & Tissue kit; Qiagen®, Hilden, Germany) according to the manufacturer’s instructions. All samples were subjected to an internal control of DNA extraction. Samples were screened for *Mycoplasma* sp. DNA by a conventional PCR protocol targeting a 384-bp fragment of the 16S rRNA gene (Table 1). The positive control was obtained from clinical samples of *M. haemocanis* from previously sequenced dog blood samples, and ultrapure water was used as a negative PCR control. The positive samples obtained were sequenced by Sanger method at Macrogen Inc. (Geumcheon-gu, Seoul, South Korea). All the sequences obtained were compared with reference sequences deposited in GenBank^®^ (https://www.ncbi.nlm.nih.gov/genbank accessed on 13 January 2021) and checked in Geneious Prime 2020.1.2 (Biomatters Ltd., Auckland, New Zealand) to ensure the quality and identity of each sequence.

### 2.3. Identification of Ectoparasites

To determine the role of ectoparasites as potential vectors of hemoplasma, ticks and fleas of dogs were identified based on morphological criteria, following taxonomic keys [31,32]. Whenever the head of the arthropod was absent, species confirmation was assessed by molecular methods (Table 1).

### 2.4. Data Analysis

All data analyses were performed in R software v. 4.0.2 (R Core. Team, Vienna, Austria). Estimated prevalence of *Mycoplasma* in each species and bioregion, and ectoparasites mean abundance and mean intensity in dogs were calculated using “epiR” package [33]. Differences in the occurrence of *Mycoplasma* sp. between species was calculated using *χ*^2^-square tests and Fisher’s exact test, and differences between overall *Mycoplasma* per bioregion were calculated using generalized linear models (GLM).

Analyses of risk factors associated with *Mhc* and *C*Mhp were calculated using generalized linear mixed models (GLMM) with binomial errors using bioregion as a random effect, to control for regional differences, using Akaike’s information criterion corrected for small sample size. *Mhc* and *C*Mhp molecular presence/absence was binary coded and compared with intrinsic (individual) variables as age and sex in foxes and in dogs, and with tick and flea abundance (defined as: none = zero ticks/fleas, low = 1–10 ticks/fleas, and high = over 10 ticks/fleas) in dogs. The best model was selected using the “dredge” function from the “MuMIn” package [34].

To infer genetic relationships among our hemoplasma sequences from dogs and foxes, we constructed median joining networks using the software PopART [35]. The obtained sequences were aligned using ClustalW in Geneious Prime 2020.1.2 (Biomatters Ltd., Auckland, New Zealand) To assess *Mhc*/*Mhf* and *C*Mhp diversity, we analyzed nucleotide polymorphisms of the 16S rRNA gene sequences using DnaSP.5 [36], obtaining haplotype diversity (Hd), nucleotide diversity (π), and the average number of nucleotide differences (k). The genetic structure was estimated through the pairwise Phi_ST_ test with 1000 permutation, performed in Arlequin v.3.5.2.2 [37], and the nearest neighbor statistic S_nn_ [38] using DnaSP.5. A network containing *Mhc*/*Mhf* sequences from dogs and the two fox species was used to infer genetic relationships among host species. To measure *Mhc*/*Mhf* genetic relationships between regions in each host, we performed network analysis with sequences from different bioregions for each host species. Network analyses containing sequences of both foxes and dogs from each bioregion was performed to evaluate genetic *Mhc*/*Mhf* and *C*Mhp diversity between host species in each bioregion. Finally, to assess hemoplasma genetic variability and structure in Chile, haplotype networks were performed between our hemoplasma sequences and those from the other two previously published studies in canids of Chile that included Darwin’s foxes and dogs [10,11].

## 3. Results

### 3.1. Hemoplasma Prevalence and Sequence Types

The screening protocol revealed *Mycoplasma* DNA in 243 dogs (observed prevalence = 38.8%, 95% confidence interval = 35.1–42.7%) and in 73 foxes (31 grey and 42 Andean foxes) (32.9%, 95% C.I. = 27.0–39.3%) (Figure 1). These differences did not differ significantly (*X*^2^ = 2.35, *p* > 0.05). Overall, *Mycoplasma* sp. prevalence in dogs across the different bioregions ranged from 32.1% in the Coastal Desert to 46.3% in the Mountain Desert (Figure 1, Table 2), whereas, in foxes, it ranged from 28.2% in the Mediterranean region to 42.1% in the Coastal Desert. The sequencing of 320 bp revealed the presence of 30 nucleotide sequence types (ntST) (Table 3), of that 24 corresponded with *Mhc*/*Mhf*. Of the remaining ntST, four corresponded to *C*Mhp, one presented 100% identity with *Candidatus* Mycoplasma haematominutum (*C*Mhm), and another one was identical to *Mycoplasma* sp. clone ZD019 (accession number MK457366), a hemoplasma detected in diverse South American carnivores (Table 3). In consequence, *Mhc*/*Mhf* and *C*Mhp DNA was found, respectively, in 23.2% and 12.8% of the dogs, and 18.8% and 7.17% of the foxes (Table 2). No difference in prevalence was found between host species for any hemoplasma species (in all cases, *X*^2^ = 0.97, *p* > 0.05). Three of the ntST were shared between dogs and foxes: two *Mhc*/*Mhf* ntST and one *C*Mhp ntST. The most prevalent one (ntST-1, *n* = 104) was shared by 81 dogs and 23 foxes.

### 3.2. Risk Factors

Overall *Mycoplasma* prevalence differed between bioregions, being significantly higher in Mountain Desert (*z*-value = 2.436, *p* < 0.05), Mediterranean (*z*-value = 1.478, *p* < 0.05), and TWR (*z*-value = 2.199, *p* < 0.05) than in Coastal Desert. Models performed in dogs indicated that *Mhc*/*Mhf* occurrence was significantly higher in males than in females (27.2% vs 18.9%, *z*-value = 2.36, *p* < 0.05; Table 4), while no other factor was associated with the occurrence *Mhc*/*Mhf*. No significant differences between bioregions were detected for *Mhc*/*Mhf*. *C*Mhp occurrence was also significantly higher in males than in females (17.8% vs. 5.6%, *z*-value = 4.02, *p* < 0.01), and in adults than in juveniles (14.7% vs. 5.5%, *z*-value = 2.43, *p* < 0.05; Table 4). *C*Mhp prevalence was significantly higher in Mountain Desert (*z*-value = 2.21, *p* < 0.05) and Steppe (*z*-value = 3.28, *p* < 0.01) than in Coastal Desert. The most prevalent tick were ticks from the *Rhipicephalus sanguineus* species group, and the most prevalent flea was *Ctenocephalides* sp. (Table 5). Neither tick nor flea abundance was significantly associated with overall *Mycoplasma* occurrence or to any of the *Mycoplasma* species in dogs. In foxes, none of the considered factors were related to the overall prevalence of *Mycoplasma* sp. nor with *Mhc*/*Mhf* or *C*Mhp (in all the cases, *p* > 0.05).

### 3.3. Genetic Relationships

**Figure 2:**
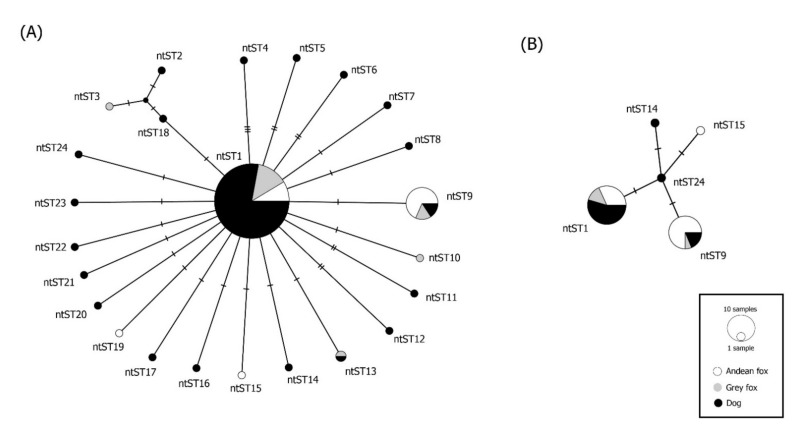
(**A**) Median-joining network of the 16S rRNA gene (322bp) of *Mycoplasma haemocanis* in rural dogs and wild foxes. (**B**) Median-joining network of the 16s gene (302bp) of *Mycoplasma haemocanis* in dogs and foxes from the Mediterranean region. The color of the circles corresponds to the species addressed. Each circle in the networks corresponds to a different nucleotide sequence type (ntST), and the size of the circles corresponds to ntST frequencies.

**Figure 3:**
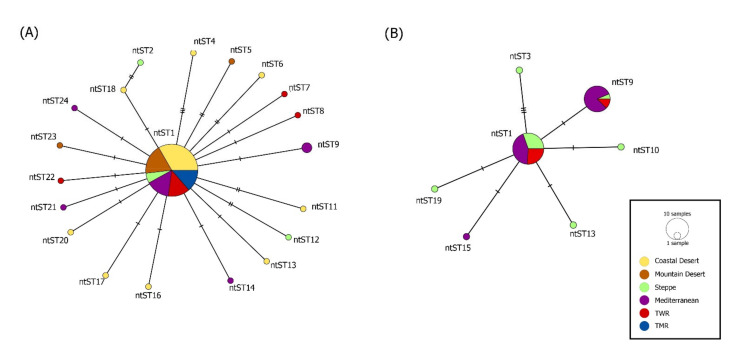
(**A**) Median-joining network of the 16S rRNA gene (322bp) of *Mycoplasma haemocanis* in rural dogs from different bioclimatic regions. (**B**) Median-joining network of the 16S rRNA gene (356bp) of *Mycoplasma haemocanis* in foxes from different bioclimatic regions. The color of the circles corresponds to the bioclimatic region where the ntST were detected. Each circle in the networks corresponds to a different nucleotide sequence type (ntST), and the size of the circles corresponds to ntST frequencies.

**Figure 4:**
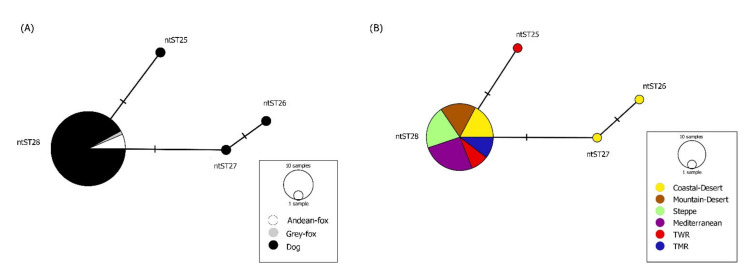
(**A**) Median-joining network of the 16S rRNA gene (312bp) of *Candidatus* Mycoplasma haematoparvum in rural dogs and wild foxes. The color of the circles corresponds to the species addressed. (**B**) Median-joining network of the 16S rRNA gene (312bp) of *Candidatus* Mycoplasma haematoparvum in rural dogs from different bioclimatic regions. The color of the circles corresponds to the species assessed or bioclimatic region were the ntST were detected. Each circle in the networks corresponds to a different nucleotide sequence type (ntST), and the size of the circles corresponds to ntST frequencies. No network exclusive of *Candidatus* Mycoplasma haematoparvum sequences from foxes is presented due to the presence of a single ntST in these animals.

Overall DNA polymorphism of sequences revealed a haplotype diversity (Hd) of 0.477 and a nucleotide diversity (π) of 0.00243 for *Mhc*/*Mhf* and Hd of 0.091 and π of 0.00039 for *C*Mhp (Table 6). The network analysis of *Mhc*/*Mhf* showed a pattern of genetic structure between dogs and Andean foxes in the country (Table 6, Figure 2A). When each bioregion was analyzed independently, genetic structure for *Mhc*/*Mhf* between species was confirmed only in the Mediterranean region (Table 6, Figure 2B). On the other hand, genetic structure of *Mhc*/*Mhf* was detected among the six bioregions both in dogs and in foxes (Table 6, Figure 3A,B). No genetic structure for *C*Mhp was detected among hosts or bioregions (Table 6, Figure 4).

Almost all the sequences obtained from dogs and Darwin’s foxes in previous studies in Chile belonged to the most prevalent ntST of our study. Only one dog (MN164349), and a Darwin’s fox (MN164353), both from Chiloé Island, presented different ntSTs. The overall Hd was 0.418 and the overall π was 0.00241. Genetic structure for *Mhc*/*Mhf* was detected between host species when these sequences were included in the analysis (Phi_ST_ = 0.1362, *p* < 0.05, S_nn_ = 0.489, *p* < 0.05; Supplementary Figure S1). The *C*Mhp sequences previously reported in Chile also belonged to the most prevalent ntSTs of the present study. The overall Hd was 0.0833 and the overall π was 0.00049. No genetic structure was detected between host species when these sequences were included in the analysis (Phi_ST_ = 0.00074, *p* > 0.05; S_nn_ = 0.917, *p* > 0.05).

## 4. Discussion

The singular geographical feature of Chile, which includes a wide range of diverse bioclimates, ranging from the hot, dry north to the cold, wet south, with high plateaus, typical Mediterranean areas, and other climates in between, allowed us to perform a unique study of the distribution of a poorly known group of pathogens both in a domestic species and wild counterparts. Some methodological issues must be considered. First, only the 16S rRNA gene was sequenced here, what prevents us to distinguish between *Mhc* and *Mhf* [39]. Nevertheless, in a recent study on Chiloé Island in Southern Chile, Di Cataldo et al. [11] confirmed by the sequencing of a portion of the RNase P gene that all these infections in dogs and foxes corresponded to *Mhc*. Moreover, *Mhf* has never been found in dogs. Therefore, we assume that most, if not all, the positive cases of the present survey correspond with *Mhc* and, for clarity, we hereafter refer to these sequences as *Mhc*. In second place, we did not evaluate the presence of possible *Mhc*-*C*Mhp coinfections. Although Soto et al. [10] did not find coinfections with hemoplasmas in dogs from southern Chile, Di Cataldo et al. [11] did confirm *Mhc*-*C*Mhp coinfections in Darwin’s foxes. Therefore, the prevalence of each species is probably underestimated. Finally, although we did not sequence the full 16S rRNA gene, we recently showed that the gene fragment sequenced here provides similar variability to the full gene sequence [40].

Other than *Mhc* and *C*Mhp, we found one grey fox of the Steppe region infected with *C*Mhm, sharing 100% identity with a sequence from a domestic cat from southern Chile, also detected in cats and guignas (*Leopardus guigna*) from several Chilean bioclimatic regions [41], but never in the bioregion found here. This hemoplasma has been anecdotally reported in dogs [7], but this is the first time it is reported in a wild canid, and probably corresponds to a spill-over from a feline species. On the other hand, the detection in an Andean fox of *Mycoplasma* sp. Clone ZD019, previously detected in Darwin’s fox, domestic cat and guignas from Chile [41], and in grey foxes from Argentina [42] suggests, as mentioned by Sacristán et al. [41], the presence of a potentially new species. This *Mycoplasma* sp. is closely related to a hemoplasma found in rodents, indicating a possibly predatory route of infection [2].

Overall *Mycoplasma* prevalence was similar between dogs and foxes across the country, being also in the range of other hemoplasma surveys published in Chile [10,11]. However, when each hemoplasma species was assessed separately, we found that the prevalence of *Mhc* in the TWR bioregion was markedly higher than reported by Soto et al. [10] in the same area. *Mhc* and *C*Mhp in dogs analyzed in our study had relatively similar prevalence’s through the six bioregions, while the network analysis indicated a geographic structure of *Mhc* both in dogs and foxes. In dogs, this may be indicating that each bioregion presents typical ntSTs, while in foxes there are two main ntSTs infecting wild canids in Chile, suggesting intraspecific transmission of this bacterium.

Intriguingly, the two most extreme bioregions studied, namely Mountain Desert and TWR, where no *R. sanguineus* species were found, showed higher *Mhc* prevalence. In the case of TWR, high *Mhc* prevalence may be associated with a high density of rural dogs in these areas [43], which facilitates the circulation of the pathogen. However, the density of dogs in the Mountain Desert is extremely low. We assume that, although the dog density in such high plateau desert is very low, dogs are very aggregated around human settlements due to the total lack of resources far from villages. Aggregation has been considered even more important than density for pathogen transmission [44]. 

The prevalence observed in foxes herein is higher when compared with other studies, i.e., 8% of grey foxes in Argentina [42], and between 1 and 4% in red foxes (*Vulpes vulpes*) worldwide [45,46,47]. This could be due to the widespread presence of free-ranging dogs in rural Chile [43], that probably are acting as reservoirs of *Mhc* for the foxes. This is supported by the fact that the two most prevalent hemoplasma ntSTs were shared between dogs and foxes throughout Chile, suggesting that cross-infection between these species is frequent. Contact with wildlife has been revealed as a risk factor for hemoplasma infection in dogs [48] although this seems not to be the case in this study. A higher *Mhc* prevalence was observed in foxes from the Mediterranean and TWR regions. As mentioned before, these two areas are inhabited by the largest number of rural free-ranging dogs in Chile [43], which may facilitate hemoplasma transmission between dogs and foxes. Although the dog density in TWR is lower, the number of natural areas where dogs and foxes could interact is high [49]. However, the average number of nucleotide differences (π) of *Mhc* sequences between the species analyzed was low, and the detected pattern of genetic structure in the Mediterranean region may be suggesting that some *Mhc* ntSTs could be enzootic in foxes in certain areas. In addition, the network analyses showed the existence of a possible founder’s effect both for *Mhc* and *C*Mhp, where one main ntST holds the majority of dog and foxes’ sequences. This suggests that these pathogens were introduced in the country with dogs and then jumped to foxes, where some variants of *Mhc* begun to circulate in an enzootic way. We also detected genetic structure for *Mhc* among the studied bioregions, suggesting that some variants are associated with some regions. This is probably due to the large distances between the studied areas.

The most prevalent ectoparasites retrieved from dogs across the country were *Rhipicephalus sanguineus* species group. and *Ctenocephalides* spp., but none of them was statistically associated with the presence of any hemoplasma species of our study. Moreover, as already mentioned, we found high prevalence in bioregions where no ectoparasites at all were detected. Previous studies already reported hemoplasmas in foxes and/or dogs in locations where *R. sanguineus* species is rare or absent [11,46,50]. This contrasts with other studies that associated the presence of *Mhc* with *R. sanguineus* species [48,51]. Altogether, there is growing evidence that canine hemotropic mycoplasmas are not only transmitted by ticks, and that alternative or concurrent transmission routes must exist. In relation to this, the higher prevalence observed (both for *Mhc* and *C*Mhp) in male dogs strongly suggests that aggressive interactions may be involved in the transmission of the bacteria through blood ingestion, as previously proposed [52]. In addition, the association of *C*Mhp with adult dogs concurs with other studies [48], indicating an increased risk of exposure to the pathogen with aging. However, none of the intrinsic factor studied here were related to *Mhc* or *C*Mhp infection in the analyzed foxes, that contrast with the higher *Mhc* prevalence observed in adult Darwin’s foxes [11]. This may indicate that different risk factors of hemoplasma infection are taking place for each fox species, that can include differences in fox and/or dog densities, suitability for ectoparasites, and behavioral components.

Hemoplasmas are characterized by being chronic infectious agents. The fact that both wild and domestics canids of Chile present a prevalence over 30% across all the different bioclimatic regions studied, emphasizes the widespread nature of the bacteria. Potential implications in the health of these animals should be addressed, having in mind that some mammals have been considered tolerant to the infection [11], while others can be seriously affected by these hemoplasmas, especially when co-infected with other pathogens [9]. It is worth noticing that some *Mycoplasma* species have also been associated with oncogenesis [4] and infection of reproductive tissues [53]. In conclusion, this country-wide survey adds substantial evidence for a little-known group of bacteria, with relevance in transmission and the identification of diverse risk factors for infection. Due to the large size of Chile, the diverse bioregions included in our study can be useful for other researchers worldwide, since they can be easily compared with many other bioregions of the world.

## Figures and Tables

**Table 1:** Genes targeted by conventional PCR and primers used in this study.

Target	Primer Names	Primer Sequences (5′–3′)	Amplicon Length (bp)	Reference
Canine endogenous control (RPS19)	RPS19F RPS19R	CCTTCCTCAAAAA/GTCTGGG1 GTTCTCATCGTAGGGAGCAAG	95	[28]
*Mycoplasma* screening (16S)	Mycop16S rRNA-F Mycop16S rRNA-R	ATGTTGCTTAATTCGATAATACACGAAA ACRGGATTACTAGTGATTCCAACTTCAA	384	[29]
Tick endogenous control (16S)	16S-F 16S-R1	TTAAATTGCTGTRGTATT CCGGTCTGAACTCASAWC	455	[30]

**Table 2:** Hemoplasma prevalence in rural dogs and foxes per bioclimatic regions in Chile.

Bioregion	Dog			Andean Fox			South American Grey Fox		
	*n*	*Mhc/Mhf*	*C*Mhp	*n*	*Mhc/Mhf*	*C*Mhp	*n*	*Mhc/Mhf*	*C*Mhp
		Prev. % (C.I.)	Prev. % (C.I.)		Prev. % (C.I.)	Prev. % (C.I.)		Prev. % (C.I.)	Prev. % (C.I.)
Coastal Desert	196	23.5 (18.1–29.9)	7.6 (4.7–12.2)	16	6.2 (0.2–28.3)	25.0 (10.2–49.5)	3	33.3 (1.7–79.2)	33.3 (1.7–79.2)
Mountain Desert	108	27.8 (20.2–36.9)	15.8 (10.1–23.8)	0	-	-	0	-	-
Steppe	75	14.7 (8.4–24.4)	22.7 (14.7–33.3)	7	28.6 (8.2–64.1)	0 (0–35.4)	41	24.4 (13.8–39.3)	12.2 (5.3–25.5)
Mediterranean	111	20.7 (14.2–29.2)	13.5 (8.4–21.1)	106	21.7 (14.9–30.5)	4.7 (2.0–10.7)	17	23.5 (9.5–47.3)	5.9 (0.3–26.7)
Temperate Warm Rainy	80	26.3 (17.9–36.8)	10.0 (5.1–18.5)	10	20.0 (5.7–50.9)	10.0 (0.5–40.4)	22	31.8 (16.4–52.7)	0 (0–14.9)
Temperate Maritime Rainy	56	25.0 (15.5–37.7)	10.7 (10.1–15.3)	0	-	-	0	-	-
Overall prevalence	626	23.8 (20.4–27.1)	12.8 (10.1–15.4)	139	20.1 (14.3–27.6)	7.2 (3.9–12.7)	83	26.5 (18.2–36.9)	8.4 (4.1–16.4)

*Mhc/Mhf*: *Mycoplasma haemocanis/Mycoplasma haemofelis*, *C*Mhp: *Candidatus* Mycoplasma haematoparvum, *n*: sample size, Prev.: Prevalence, C.I.: 95% Confidence Intervals.

**Table 3:** *Mycoplasma* sp. 16S rRNA sequences types detected in rural dogs and foxes in Chile.

ntST	Host (*n*)	Bioregion	P.I.	Best GenBank^®^ Match
ntst1	Dog (81), Andean fox (9), South American grey fox (14)	All bioregions	100%	*M. haemocanis*, dog from Chile (KY117653)
ntst2	Dog (1)	Steppe	99.2%	*M. haemofelis*, cat from China (MH447082)
ntst3	South American grey fox (1)	Steppe	99.2%	*M.a haemofelis*, cat from China (MH447082)
ntst4	Dog (1)	Coastal Desert	99.2%	*M. haemofelis*, cat from China (MH447082)
ntst5	Dog (1)	Mountain Desert	99.4%	*M. haemofelis*, cat from China (MH447082)
ntst6	Dog (1)	Coastal Desert	99.4%	*M. haemofelis*, cat from China (MH447082)
ntst7	Dog (1)	TWR	99.7%	*M. haemofelis*, cat from China (MH447082)
ntst8	Dog (1)	TWR	99.7%	*M. haemofelis*, cat from China (MH447082)
ntst9	Andean fox (13), Dog (3), South American grey fox (3)	Steppe, Mediterranean and TWR	99.7%	*M. haemofelis*, cat from China (MH447082)
ntst10	South American grey fox (1)	Steppe	99.7%	*M. haemofelis*, cat from China (MH447082)
ntst11	Dog (1)	Coastal Desert	99.4%	*M. haemofelis*, cat from China (MH447082)
ntst12	Dog (1)	Steppe	99.5%	*M. haemofelis*, cat from China (MH447082)
ntst13	Dog (1), South American grey fox (1)	Coastal Desert and Steppe	100%	*M. haemocanis*, dog from Portugal (GQ129118)
ntst14	Dog (1)	Mediterranean	99.7%	*M. haemofelis*, cat from China (MH447082)
ntst15	Andean fox (1)	Mediterranean	100%	*M. haemofelis*, cat from China (MH447082)
ntst16	Dog (1)	Coastal Desert	99.7%	*M. haemofelis*, cat from China (MH447082)
ntst17	Dog (1)	Coastal Desert	99.7%	*M. haemofelis*, cat from China (MH447082)
ntst18	Dog (1)	Coastal Desert	99.7%	*M. haemofelis*, cat from China (MH447082)
ntst19	Andean fox (1)	Steppe	99.7%	*M. haemofelis*, cat from China (MH447082)
ntst20	Dog (1)	Coastal Desert	99.7%	*M. haemofelis*, cat from China (MH447082)
ntst21	Dog (1)	Mediterranean	99.7%	*M. haemofelis*, cat from China (MH447082)
ntst22	Dog (1)	TWR	99.7%	*M. haemofelis*, cat from China (MH447082)
ntst23	Dog (1)	Mountain Desert	99.7%	*M. haemofelis*, cat from China (MH447082)
ntst24	Dog (1)	Mediterranean	99.7%	*M. haemofelis*, cat from China (MH447082)
ntst25	Dog (1)	TWR	99.4%	*C.* M. haematoparvum, dog from Chile (KY117661)
ntst26	Dog (1)	Coastal Desert	99.7%	*C.* M. haematoparvum, dog from Chile (KY117661)
ntst27	Dog (1)	Coastal Desert	99.7%	*C.* M. haematoparvum, dog from Chile (KY117661)
ntst28	Dog (58), Andean fox (4), South American grey fox (1)	All bioregions	99.7%	*C.* M. haematoparvum, dog from Chile (KY117661)
ntst29	South American grey fox (1)	Steppe	100%	*C.* M. haemominutum, cat from Chile (MN543625)
ntst30	Andean fox (1)	Coastal Desert	100%	100% *Mycoplasma* sp. clone ZD019, *Lycalopex fulvipes* from Chile (MK457366)

ntST: nucleotide sequence type, P.I.: Percentage of identity by BLAST^®^ analysis, TWR: Temperate Warm Rainy.

**Table 4:** Best GLMM representing multivariate relationships between predictor variables and detection of *Mycoplasma haemocanis/Mycoplasma haemofelis* and *Candidatus* Mycoplasma haematoparvum in free-ranging rural dogs.

	Estimate ± SE	*Z* Value	AIC	Deviance	Df
***Mhc/Mhf***			670.8	662.8	605
(Intercept)					
Sex male	0.475 ± 0.2	2.366*			
					
***C*Mhp**					
(Intercept)			443.3	429.3	602
Age juvenile	−1.014 ± 0.4	−2.428*			
Sex male	1.252 ± 0.3	4.016**			
Low flea infestation	1.253 ± 1.0	1.186			
No flea infestation	1.688 ± 1.03	1.629			

*Mhc/Mhf*: *Mycoplasma haemocanis/Mycoplasma haemofelis*, *C*Mhp: *Candidatus* Mycoplasma haematoparvum. Bioregions were assigned as random effect, to control for regional differences. * *p*-value < 0.05; ** *p*-value < 0.01.

**Table 5:** Overall observed prevalence, mean abundance, and mean intensity of ectoparasites on dogs in Chile.

Ectoparasite	*n*	Prev. (95% C.I.)	M.A. ± S.E.	M.I. ± S.E.
Overall ticks	1208	27.2 (23.8–30.9)	1.9 ± 0.2	7.3 ± 0.7
*Rhipicephalus sanguineus* species group	1198	26.9 (23.5–30.5)	1.9 ± 0.2	7.3 ± 0.7
*Amblyomma tigrinum*	10	0.96 (0.4–2.0)	0.01 ± 0.009	1.7 ± 0.7
Overall fleas	1513	32.6 (29.0–36.5)	2.4 ± 0.2	7.4 ± 0.7
*Ctenocephalides* sp.	790	24.2 (20.9–27.6)	1.3 ± 0.1	5.2 ± 0.4
*Pulex irritans*	385	17.1 (14.3–20.3)	0.6 ± 0.1	3.6 ± 0.4
*Echidnophaga gallinacea*	338	3.0 (1.8–4.6)	0.5 ± 0.2	16.2 ± 3.7

*n*: number of sampled animals, Prev.: prevalence, C.I.: Confidence interval, M.A.: Mean abundance, S.E.: Standard estimate, M.I.: mean intensity.

**Table 6:** Genetic diversity and polymorphism of the 16S rRNA gene sequences of *Mycoplasma haemocanis*/*Mycoplasma haemofelis* and *Candidatus* Mycoplasma haematoparvum detected in different canid hosts in Chile.

*Mycoplasma* Species	Bioregion	Host	Hd	π	K	S_nn_	Phi_ST_	*p*-Value
*Mhc/Mhf*	All	All	0.477	0.0024	0.6380	0.6026	0.1663	< 0.01
		Dog	0.370	0.0019	0.5083			
		Grey fox	0.505	0.0029	0.7684			
		Andean fox	0.587	0.0026	0.6848			
		Only dogs	0.325	0.0015	0.3949	0.2250	0.1863	< 0.05
		Only foxes	0.638	0.0029	0.8664	0.4507	0.0956	< 0.05
	Steppe	All	0.614	0.0035	1.2398	0.3445	0.0160	> 0.05
		Dog	0.524	0.0040	1.4286			
		Grey fox	0.666	0.0034	1.2000			
		Andean fox	1.0	0.0028	1.0000			
	Mediterranean	All	0.567	0.0019	0.6304	0.5475	0.3553	< 0.01
		Dog	0.419	0.0014	0.4559			
		Grey fox	0	0	0			
		Andean fox	0.542	0.0019	0.6144			
	TWR	All	0.462	0.0016	0.5146	0.5014	0.1461	> 0.05
		Dog	0.423	0.0014	0.4615			
		Grey fox	0.500	0.0015	0.5000			
		Andean fox	1.0	0.0031	1.000			
*C*Mhp*	All	All	0.909	0.0004	0.1221	0.8810	0.0083	> 0.05
		Dog	0.097	0.0004	0.1301			
		Andean fox	0	0	0			
		Only dogs	0.097	0.0004	0.1301	0.1701	0.0112	> 0.05

*CMhp analyses in grey foxes were not performed (only one *C*Mhp sequence available). Bioregions with insufficient number of sequences are not shown in the table. *Mhc/Mhf*: *Mycoplasma haemocanis/Mycoplasma haemofelis*, *C*Mhp: *Candidatus* Mycoplasma haematoparvum. Hd: Haplotype diversity, π: nucleotide diversity, K: nucleotide difference number.

## Data Availability

The data supporting the conclusions of this article are included within the article. All new obtained sequences were submitted to GenBank^®^ under the accession numbers MW629055-MW629079.

## Supplementary Materials

The following are available online at https://www.mdpi.com/article/10.3390/microorganisms9050919/s1, Supplementary Table S1: Main characteristics of the bioclimatic regions addressed in this study; Figure S1: Median-joining network of the 16s gene (384bp) of *Mycoplasma haemocanis* in rural dogs and wild foxes from our study and from previously published sequences by other authors.
