# Supplementary material for: Widespread Infection with Hemotropic Mycoplasmas in Free-Ranging Dogs and Wild Foxes Across Six Bioclimatic Regions of Chile
Source: Microorganisms. 2021 Apr 24;9(5):919. doi: 10.3390/microorganisms9050919 (PMC8145368; doi:10.3390/microorganisms9050919)

Figure S1. Median Joining Network of the 16s gene (384bp) of Mycoplasma haemocanis in rural dogs and wild foxes from our study and from previously published sequences by other authors. The color of the circles corresponds to the species addressed; the size of the circles corresponds to ntST frequencies.

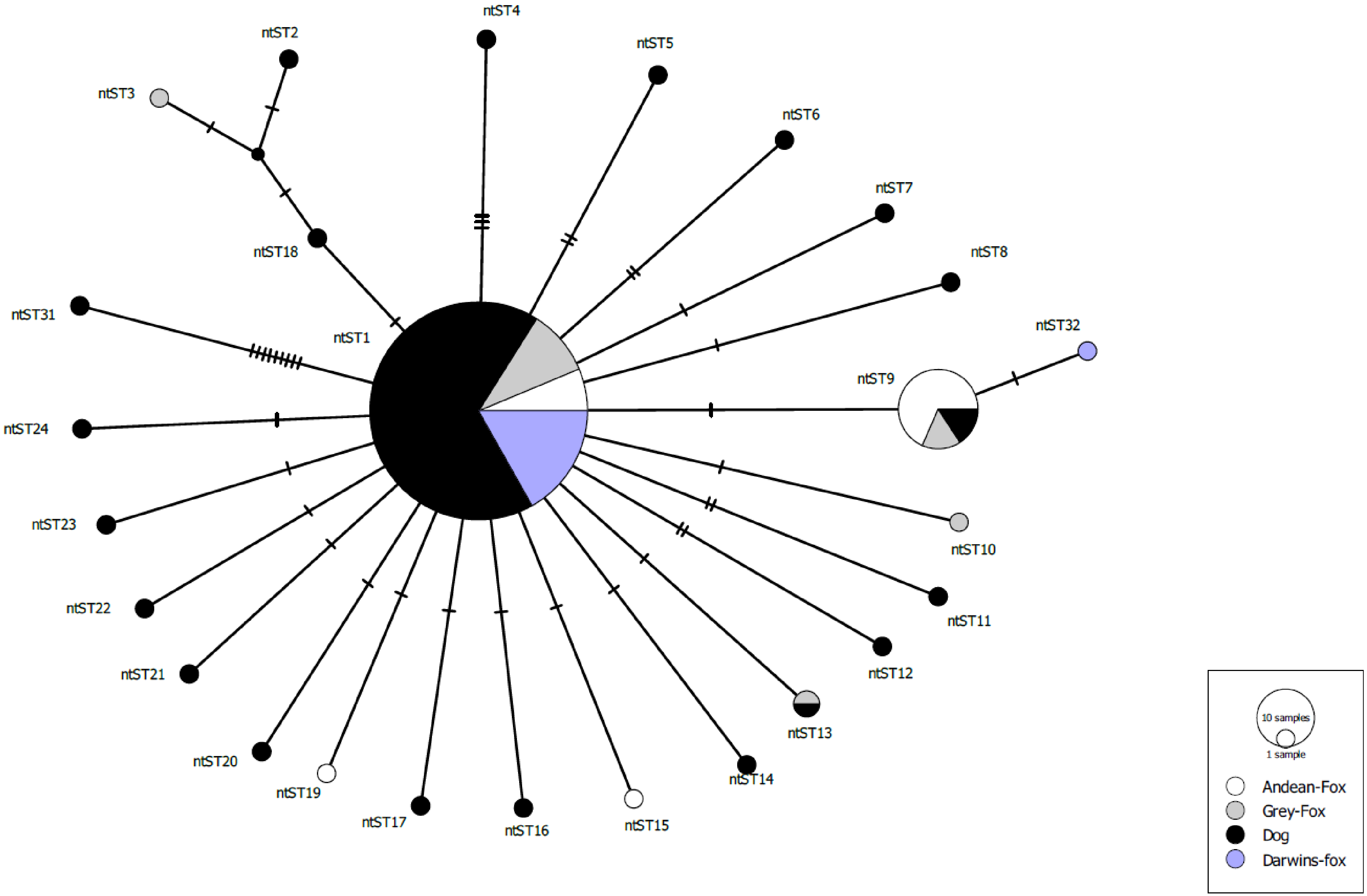

Supplement: Supplementary file 1 [file microorganisms-09-00919-s001.zip › Supplementary Figure 1.pdf]
