# Supplementary material for: Widespread Infection with Hemotropic Mycoplasmas in Free-Ranging Dogs and Wild Foxes Across Six Bioclimatic Regions of Chile
Source: Microorganisms. 2021 Apr 24;9(5):919. doi: 10.3390/microorganisms9050919 (PMC8145368; doi:10.3390/microorganisms9050919)
Supplement: Supplementary file 1 [file microorganisms-09-00919-s001.zip › Supplementary Table 1.pdf]

Table S1. Main characteristics of the bioclimatic regions addressed in this study.

Bioclimatic region	Mean rainfall in warmer seasons	Mean temperature in warmer seasons	Mean rainfall in colder seasons	Mean temperature in colder seasons	Mean annual relative humidity	Dog abundance
Coastal desert	Less than 1mm	21ºC	Less than 1mm	15.6°C	70%	Medium
Mountain desert	Less than 1mm	19.9ºC	13mm	11.8°C	74%	Low
Steppe	22 mm	26.4ºC	87mm	25.4°C	80%	Low
Mediterranean	3 mm	19.9ºC	82mm	8.6°C	75%	High
Temperate warm rainy	13 mm	19.3ºC	175mm	8.2°C	76%	Medium
Temperate cold rainy	120 mm	14.1ºC	286mm	7.1°C	82%	Low

Dog abundance was grouped according the number of dogs per km^2 as: low (0-4 dogs/km^2), medium (5-16 dogs/km^2) and high (17-30 dogs/km^2). Information was provided by Mann (1960), CONAMA (2008), INE (2014), and Astorga et al. (2015).

1. Mann, G. Regiones biogeográficas de Chile. *Investig. Zoológicas Chil.* **1960**, *6*, 15–49.
2. INE, I.N. de E. *Compendio Estadístico 2014*; 2014; ISBN 9789563231526.
3. Astorga, F.; Escobar, L.E.; Poo-Muñoz, D.A.; Medina-Vogel, G. Dog ownership, abundance and potential for bat-borne rabies spillover in Chile. *Prev. Vet. Med.* **2015**, *118*, 397–405, doi:10.1016/j.prevetmed.2015.01.002.
4. CONAMA *Biodiversidad de Chile: Patrimonio y desafíos*; 2nd ed.; 2008;
